# Physiological effects of different stubble height and freeze-thaw stress on *Secale cereale* L. seedlings

**DOI:** 10.1186/s12870-021-03235-8

**Published:** 2021-10-06

**Authors:** Longtian Zhang, Guozhang Bao, Mengyu Zhang, Zihang Yu, Tao Guan, Jingwen Li, Yidan Su, Jinghui Xi

**Affiliations:** 1 Key Laboratory of Groundwater Resources and Environment of the Ministry of Education (Jilin University), Jilin Provincial Key Laboratory of Water Resources and Environment; College of New Energy and Environment, Jilin University, Changchun, 130012 China; 2 College of Plant Science, Jilin University, Changchun, 130062 China

**Keywords:** Freeze-thaw stress, Stubble height, Osmotic adjustment, *Secale cereale* L

## Abstract

**Background:**

As a biennial plant, *Secale cereale* L is usually harvested in the autumn in the northern part of China where the temperature difference between day and night is of great disparity Through the pot experiment, the seedlings were cut to 2, 6 and 10 cm stubble height, and the simulated freeze-thaw (FT) stress (10/− 5 °C) was carried out after 6 days regrowth. The physiological effects of FT with different stubble height were revealed by analyzing the relative water content (RWC), osmotic adjustment substance concentration (soluble sugar and protein), membrane peroxidation (MDA) and catalase (CAT) activity.

**Results:**

The results demonstrated that under freeze stress (− 5 °C), the content of soluble protein and MDA decreased and the seedlings of 2 cm treatment kept higher level of soluble protein and MDA, while the seedlings of 6 and 10 cm treatments kept higher level of the RWC, soluble sugar content, and CAT activity. After FT stress, the content of soluble sugar and protein, RWC in the 6 cm treatment were higher than those in 2 cm and 10 cm treatments, and the CAT activity in 10 cm treatment was the highest while the MDA content is lower.

**Conclusion:**

These data suggest that keeping high stubble height is more adaptive for short-term FT stress.

## Background

*Secale cereale* L, as one of the most important crops in the Triticeae for nutrition and feed in North America, Europe and East Asia, is known to be of tremendously tolerance to diverse environmental stresses, including drought and frost [15]. Sown in late summer or early autumn, winter forage usually won’t be harvested until midsummer. The long term growth means that the crops will possess longer periods of time for growing, sunlight and water use, which contributes to higher yields than spring crops. In general, in order to ensure the quality and yield of overwintering forage, folks usually choose to mow in late autumn before wintering [25].

Mowing and harvesting can eliminate senescent tissues of plant, maintain the high growth vitality of leaves, stimulate the compensatory growth of plants, and influence the nutrition, which act as an efficient way to rapidly improve the crop quality. Additionally, mowing enhances the nutrient content of barley at the tillering stage more than the grain filling stage. Additionally, stubble height is also a significant factor affecting forage yield and quality [16]. Mowing and harvesting will trigger the anti-oxide enzyme system and promote osmoregulatory substances of the stubble. During the compensatory growth, CAT will be able to remove the reactive oxygen species of the leaves timely, maintaining a relatively low-level cell membrane peroxidation and integrality. Accumulated osmoregulatory substances hold the water balance of cells. Therefore, the anti-oxide enzymes and the osmoregulatory substances act as a significant role in healing the wound and accelerating the compensatory growth [8]. The ability to regenerate would vary depending on different stubble height, causing discrepancy in the accumulation and biomass between plants [7]. Mild mowing promotes plant regeneration and compensatory growth [28]. Plants with higher stubble height tend to accumulate more nutrients than the lower one [27]. Harvest at a low cutting height will remove the majority, even the whole, photosynthetic tissues and some of the stem tissue containing nonstructural carbohydrates, reducing the energy sources for regrowth [2]. It was found that increasing stubble height in hot summer could improve the regeneration ability of forage. Not only effects cutting height the quality of forage, but also directly determine the overwintering ability of plant s[14]. Crops cut in late September were found to have a superior carbohydrate accumulation through leaf regrowth, leading to an advantageous winter hardiness and subsequent regrowth [21].

Apart from tropical zones, rarely nowhere else on earth could avoid sub-zero temperature, which makes freezing damage a global concern (Bredow and Walker 2017 [6]). The temperature stress imposed on plants has a huge impact on agriculture [3]. In high latitudes, the great distinct temperature between day and night in early spring and late autumn makes plants in this area suffer from freeze-thaw (FT) stress during the growth process, resulting in a large amount of crop losses [17]. In winter, forage is subjected to FT stress and cold stress enhances the production of reactive oxygen species (superoxide anion radical, hydrogen peroxide and hydroxyl radical) in cellular compartments such as chloroplasts, peroxisomes, and mitochondria [33]. In addition, chilling stress leads to water deficits by damaging cell membranes. In order to reduce the damage to plants under FT stress, osmoregulatory substances such as soluble protein and soluble sugar accumulate in plant tissues to maintain the osmotic pressure of cells [22]. At the same time, when exposed to low temperature stress, the increase of reactive oxygen species in the plant may cause the changes in density and activity of antioxidant enzymes to reduce the damage of active oxygen to plants [12].

While the previous studies have explored the effects of stubble height or low temperature on *Secale cereale* L, little is known about the combined effect of FT stress and stubble height on it. This paper is focused on the physiological response to FT stress and stubble height of *Secale cereale* L by evaluating the changes of osmotic substance contents, relative water content (RWC) and catalase (CAT) activity, which provides a unique angle into the forage overwinter and cold resistance. These findings will contribute to the range management.

## Result

### Changes in relative water content

**Fig. 1:**
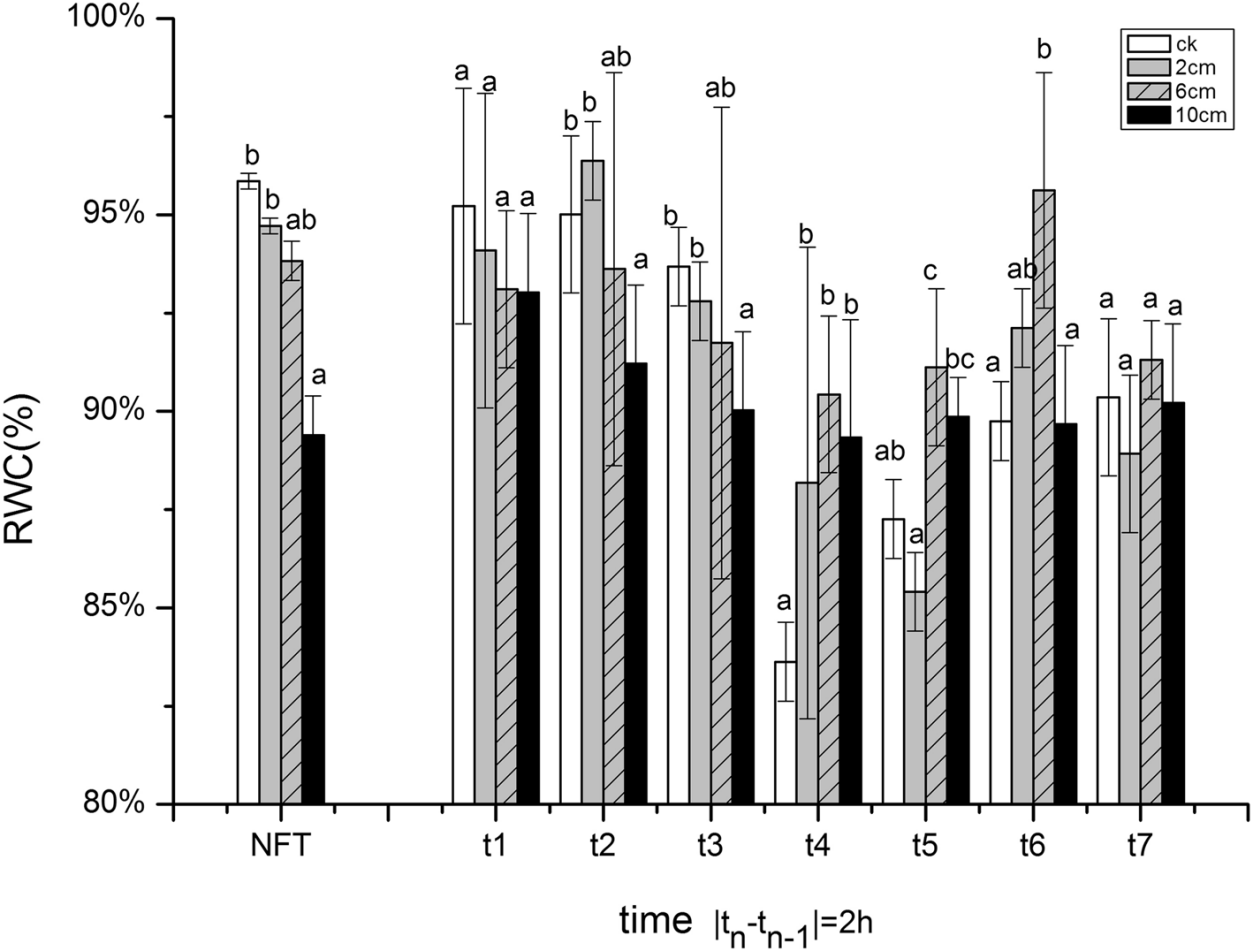
The effect of stubble height and FT stress on the RWC in seedlings. Rye was cut to 2 cm, 6 cm, 10 cm and no cutting (CK group), and FT stress management after 6 d regrowth. The sample was taken once every 2 h, and the corresponding temperature gradient was at 10, 5, 0, − 5, 0, 5 and 10 °C. No freeze-thaw (NFT) samples taken at the same time. The error bars indicate SE [t1 ~ t7 (*n* = 3); NFT (*n* = 21)]. Bars not sharing the same letter are significantly different. (*P* < 0.05)

Compared with NFT, the RWC of the seedlings decreased as the temperature dropped to − 5 °C. During t1 ~ t3 the RWC was higher in 2 cm treatment seedlings with the temperature dropping at first. But when the temperature dropped to - 5 °C (t4), the RWC of the 6 cm treatment was higher than other treatments. In the thawing stage, the RWC of seedlings was positively related with the rising temperature, and the 6 cm treatment was significantly higher than other treatments at t6. After FT stress (t7), there were no significantly differences about the RWC values among the treatments, but the RWC of 6 cm treatment was higher than 2 cm and 10 cm ones. (Fig. 1).

### Changes in soluble sugar and soluble protein contents

**Fig. 2:**
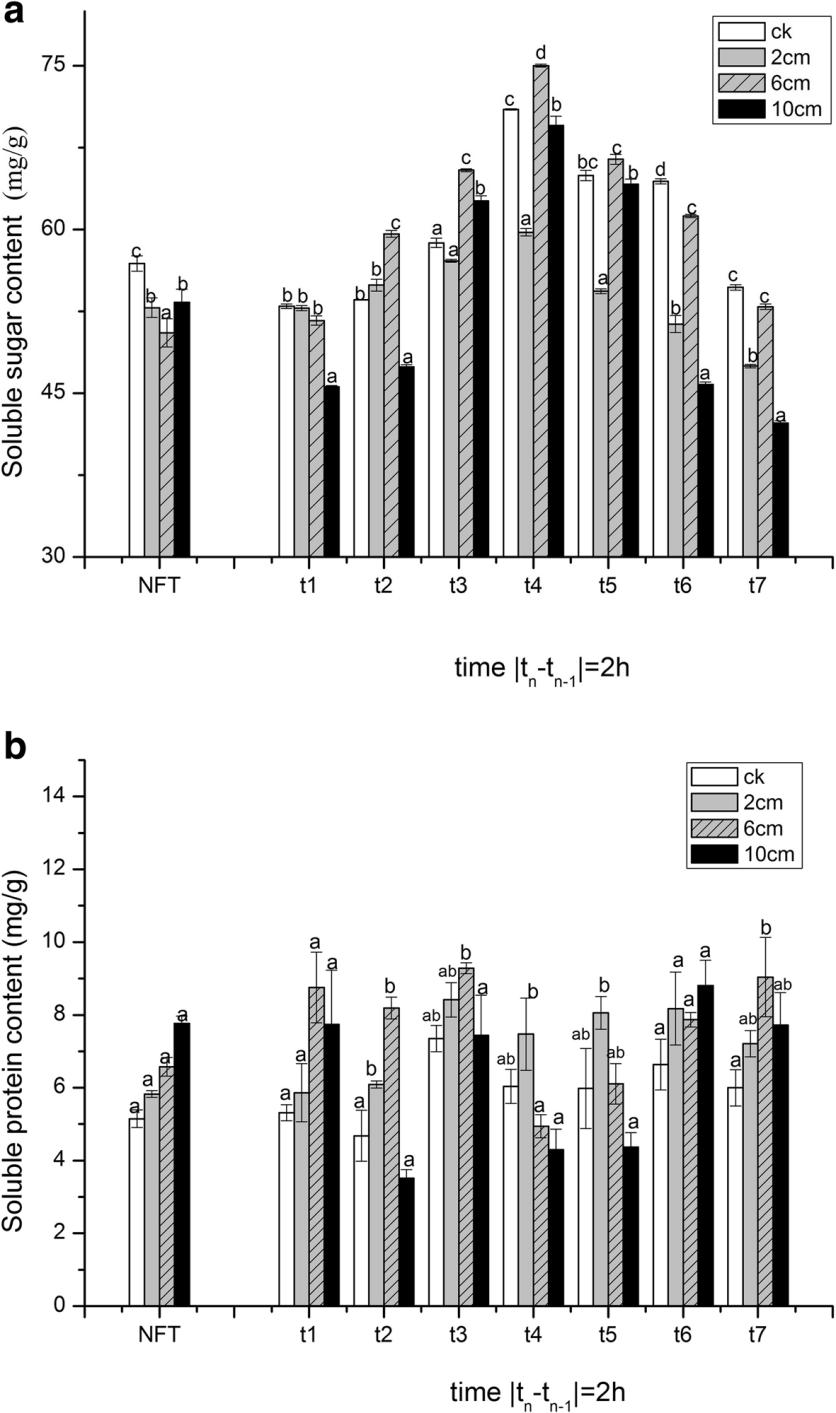
The effect of stubble height and FT stress on the content of soluble sugar (**a**) and soluble protein (**b**) in seedlings. Rye was cut to 2 cm, 6 cm, 10 cm and no cutting (CK group), and FT stress management after 6d regrowth. The sample was taken once every 2 h, and the corresponding temperature gradient was at 10, 5, 0, − 5, 0, 5 and 10 °C. No freeze-thaw (NFT) samples taken at the same time. The error bars indicate SE [t1 ~ t7 (*n* = 3); NFT (*n* = 21)]. Bars not sharing the same letter are significantly different. (*P* < 0.05)

To determine the changes of cell osmotic material, the contents of soluble protein and soluble sugar were measured. At the freezing stage (t1 ~ t4), the content of soluble sugar increased as the temperature decreased and soluble sugar accumulation appears to be higher than that of NFT at t4. The 6 cm treatment accumulated more soluble sugar than 2 cm and 10 cm treatments at t2 ~ t4. At the thawing stage, in all treatments the content of soluble sugar decreased with the rise of temperature (Fig. 2a). With the temperature decreasing, the soluble protein content at t4 was lower than that at t1, and there were more accumulating in 2 cm treatment than in 6 cm and 10 cm treatments at the same time. Data also showed that soluble protein content increased with temperature rise, and the content in 2 cm treatment was the minimum in the cutting treatments at t7. (Fig. 2b).

### Changes in lipid peroxidation

**Fig. 3:**
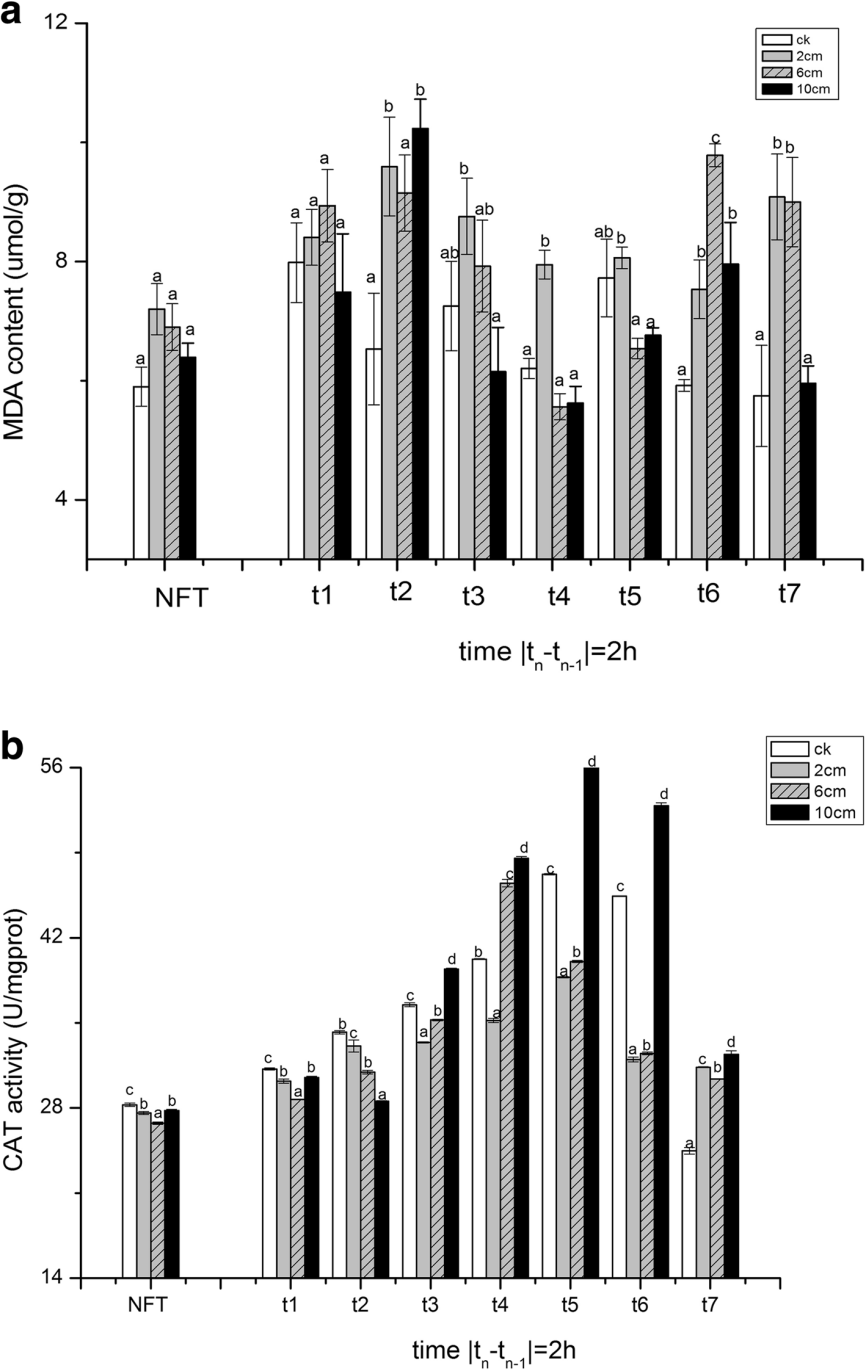
The effect of stubble height and FT stress on the MDA content (**a**) and CAT activity (**b**) in seedlings. Rye was cut to 2 cm, 6 cm, 10 cm and no cutting (CK group), and FT stress management after 6d regrowth. The sample was taken once every 2 h, and the corresponding temperature gradient was at 10, 5, 0, − 5, 0, 5 and 10 °C. No freeze-thaw (NFT) samples taken at the same time. The error bars indicate SE [t1 ~ t7 (*n* = 3); NFT (*n* = 21)]. Bars not sharing the same letter are significantly different. (*P* < 0.05)

Malondialdehyde content reflects the degree of plant cell membrane peroxidation. MDA content increased with the temperature decreasing to 5 °C (t2) but when the temperature continued to drop, the MDA content decreased at t3 ~ t4. It can be observed that as the temperature increased, the seedlings at 5 °C accumulated more MDA than at − 5 °C during thawing stage. The MDA content in seedling of 2 cm treatment was significantly higher than that in other treatments at t4. After FT stress (t7), 6 cm and 2 cm treatments still kept high MDA content compared with NFT (Fig. 3a).

### Changes in catalase activity

CAT is an antioxidant enzyme. The CAT activity increased with the temperature rise in all treatments. Seedlings of the 10 cm treatment kept higher CAT activity than the 2 and 6 cm ones at t4 (− 5 °C). The CAT activity still increased when the temperature rised at thawing stage, and seedlings of 10 cm treatment always had higher CAT activity than other cutting treatments t4 ~ t7. After FT stress, the CAT activity demonstrated lower than it was at t6 in all treatments at t7. (Fig. 3b).

## Discussion

The leaf RWC is a worthwhile indicator for determining the water status of plant leaves under cold stress [13]. We observed that the RWC in all treatments was lower than that in NFT when temperature dropped to − 5 °C, which was in accordance with the results observed by Yildiztugay, who found that there was a slight reduction in RWC in the plants exposed to the lowest cold stress and dropped to the minimum [33]. Plants exposed to chilling stress often show water-stress symptoms because of chilling induced inhibition of water uptake and water loss [11]. The reduction in leaf RWC in cold-stressed leaves was suggestive of the development of water stress [18]. In this study, under FT stress, the RWC of cutting treatments decreased more than CK group, and 6 cm treatment had a higher RWC than other cutting treatments. After FT stress, the 6 cm treatment can maintain stable RWC, indicating that different stubble height can affect the seedlings RWC changes under FT stress. Compared with lower stubble height, the higher ones were more beneficial to subsequent regrowth and overwinters in November [24].

Cellular dehydration is one of the most damaging consequences of freezing, metabolism is redirected towards the production of low molecular weight cryoprotectants, such as soluble sugar, and begin to synthesis more soluble protein [19]. Increases in some soluble sugars and proteins are some of the most important metabolic defenses against freezing stress [18]. We observed that soluble sugar and soluble protein contents at t3 were higher than those at t1 as temperature dropped. Some freeze-tolerant plants mitigate the risk of frozen by expressing anti-freezing proteins [9]. A study of the response of grapes in low temperature stress found that the carbohydrates in the plants increased as the temperature decreased [29], probably due to the osmotic adjustment by synthesis of carbohydrates to counteract cellular dehydration and turgor loss to alleviate the damages [22]. But the soluble protein content decreased at t4 compared with t3. In previous study, plant consumed too much soluble protein at low temperatures to maintain cell stability which caused the content of soluble protein decreased [5]. The soluble protein content of 2 cm treatment was higher than others at t4 and t5 under freeze stress. That may be because the nutrients were reused and transferred from the leaves to the rhizomes. The low retention height has a greater regenerative advantage, leading more nutrients to be accumulated [16]. Although it has been previously reported that as stubble height increased, soluble sugar content increases and helps reduce cell dehydration due to temperature changes [32], we found that 6 cm treatment accumulated more soluble sugar and soluble protein than 2 and 10 cm after FT stress. It seemed that increasing the stubble height within a range was beneficial to osmotic adjustment substance under FT stress.

Chilling stress is associated with ROS accumulation that can trigger membrane unsaturated fatty acids peroxidation, which can be assayed by MDA content measurement [30]. As observed in MDA content, freezing stress induced the accumulation of MDA contents. Cold inducing oxidative damage in both root and leaf tissues of tomato were demonstrated in terms of accumulating MDA levels [29]. But as the temperature dropped, compared to the t3, MDA content of each cutting treatments was lower at t4, and the activity of CAT increased at the same time (Fig. 3b). Aghdam found that after being treated with salicylic, the antifreeze property of the plant was enhanced, the activity of antioxidant enzyme was increased and the content of MDA decreased [1]. Low temperature causes an increase in free radicals in the cells, activates the antioxidant system, and increases the activity of the CAT enzyme, thereby alleviate the peroxidation in the plant [31]. The result indicated that the MDA content in 2 cm treatment was higher than those in 6 cm and 10 cm ones. After FT stress, 10 cm treatment kept a higher CAT activity and lower MDA content compared with other treatments. For the reason that higher stubble height might have better frost resistance compared with lower ones. New tissues are usually more metabolically and enzymatically active but shorter stubble during cutting treatments resulted in decreased root length and weight, inhibiting the transport of nutrients, water, and hormones in the shoot tissue [20].

## Conclusions

The physiological effects of FT stress on different stubble height varied. The damage of 6 and 10 cm treatments was less than that of 2 cm one after FT stress, which provides a reference for the selection of reasonable stubble height before winter in the high latitude area. However, considering that the duration of artificial FT stress of our experiment was relatively short and the possible snow cover on the forage during overwintering in the field, more comprehensive experiments are expected to be carried out to find alternative reasonable stubble heights.

## Methods

### Plant materials

Approximately 1000 seeds of Dongmu-70 provided by Ditong Seed Company of China were selected, soaked in 0.1% acidic KMnO_4_ solution for 2 h, and then rinsed with distilled water. The seeds were then arranged onto trays and covered with two layers of filter paper, and 100 ml of Hoagland nutrient solution was added. After the seeds germinated under dark conditions at 20 °C for 24 h in an MGC-450BP light incubator (Shanghai Yiheng Scientific Instruments Co., Ltd), full and similarly sized seedings were selected, placed on trays of 26 × 18 cm (length × width; 23 lines × 40 seeds per line) and then transferred to an MGC-450BP light incubator (Shanghai Yiheng Scientific Instruments Co., Ltd) under 12-h light (16,500 lx; 25 °C)/12-h dark (0 lx; 15 °C) conditions for 1 week. 80 ml of nutrient solution was given every day – 40 ml each morning and evening.

### Plant growth and stress treatment

Seeds of *Secale cereale* L were surface sterilized in aqueous solutions of KMnO_4_ (0.1%) and washed in deionized water till the water clear. Sterilized seeds were soaked for 4 h in deionized water and then laid in the grid layer of the cultivating tray (34 × 25 × 12 cm). We added the Hoagland nutrient solution to the bottom of the tray, and then covered the transparent lid. Seeds were incubated in a growth chamber with the following conditions: photoperiod 12/12 h (day/night), daily temperature 25/20 °C. The unspotted seeds were picked out on the second day. The lid of the cultivating tray was removed when the seedlings grew to 3 cm and we added with 500 ml Hoagland nutrient solution every 2 days. After 6 days of cultivation, when the average height of seedling was 15 cm, clipped the seedlings to 2, 6, 10 cm height and no clipping (CK treatment) and kept to regrow in the same conditions for 6 days. Seedlings were then transferred to a BPHJ-120A-type alternation refrigerator for the FT processes. For the freeze stress treatment, the temperature was lowered by 5 °C every 2 h. Similarly, for the thaw stress treatment, the temperature was raised by 5 °C every 2 h. The rate of temperature change was 0.5 °C / 12 min. We took the sample every 2 h, and the corresponding temperature gradient was at 10, 5, 0, − 5, 0, 5 and 10 °C (the corresponding sampling time was t1 ~ t7), after which the sample was immediately wrapped with foil paper, fixed in liquid nitrogen for 50 s, stored at − 70 °C, no freeze-thaw (NFT) samples taken at the same time.

### Biochemical characterization

Relative water content (RWC) of leaf was measured according to Kamrun Nahar [26]. Fresh weight (FW), turgid weight (TW) and dry weight (DW) of seedlings were taken; RWC was calculated by following formula:

$$\mathrm{RWC}\left(\%\right)=\left(\mathrm{TW}-\mathrm{FW}\right)/\left(\mathrm{TW}-\mathrm{DW}\right)\times 100\%$$

Soluble protein content was analyzed using method adapted from Liu [23]. Seedling tissue (0.1 g) with 5 ml deionized water was ground into homogenate and then centrifuged at 3000 r/min for 10 min. 1 ml of supernatant was took and put into the test tube and then 4 ml deionized water was added. After dilution, 1 ml diluent was extracted, 5 ml coomath brilliant blue g-250 solution was added. The solution was shook well and was let stand for 2 min. The absorbance was determined at the wavelength of 595 nm. The soluble protein content was determined by protein standard curve.

Sulfuric acid anthrone was used to measure the content of the soluble sugar as previously described [4]. 0.1 g of fresh sample was taken and put into a large test tube. 15 ml distilled water was added and boiled in a boiling water bath for 20 min. 100 ml volumetric flask was removed, cooled and filtered, the residue was rinsed with distilled water for several times, and the volume was scaled. The extract solution of the sample to be tested was 1.0 ml, added with 5 ml of anthrone reagent, shaken well, and boiled in a boiling water bath for 10 min, then removed and cooled. The absorbance was measured at 620 nm.

The level of lipid peroxidation was measured by estimating malondialdehyde (MDA), using thiobarbituric acid (TBA) [10]. We added 5 ml TCA to 0.5 g sample and ground them into homogenate. After centrifugation, removed 2 ml of supernatant, added 2 ml TBA, mixed, bath at 95 °C for 25 min, and then cooled to room temperature, reading at a wavelength of 450, 532 and 600 nm. MDA content was calculated by formula:

$$\mathrm{MDA}\ \mathrm{content}\ \left(\mu \mathrm{mol}/\mathrm{l}\right)=6.45\ \left({\mathrm{D}}_{532}-{\mathrm{D}}_{600}\right)-0.56{\mathrm{D}}_{450}$$

Assay kits produced by the Nanjing Jian Cheng Bioengineering Institute (7200, UNICO, Shanghai, China) were used to determine the activity of catalase (CAT).

### Data processing

Statistical analysis was performed with R 3.3.1 statistical software (R Foundation for Statistical Computing, Vienna, Austria). ANOVA was used to determine treatment differences at each time point. Individual differences among means were determined by Duncan’s test at *P* < 0.05.

## Data Availability

All data generated or analyzed during this study are included in this published article and its. supplementary information files.

## Abbreviations

FT: Freeze-thaw
RWC: Relative water content;
MDA: Malondialdehyde
CAT: Catalase
SP: Soluble protein
TBA: Thiobarbituric acid
TCA: Tricarboxylic acid
FW: Fresh weigh
TW: Turgid weight
DW: Dry weight
NFT: No freeze-thaw
